# Clinical and radiographic evaluation of silver diamine fluoride versus mineral trioxide aggregate as indirect pulp capping agents in deeply carious first permanent molars a randomized clinical trial

**DOI:** 10.1038/s41405-024-00286-5

**Published:** 2025-01-09

**Authors:** Marwa Ahmed Ahmed Zaghloul, Manal Ahmed El Sayed, Randa Youssef Abd Al-Gawad, Ahmed Mohamed Abd El- Samad

**Affiliations:** 1 Pediatric Dentistry and Dental Public Health, Faculty of Dentistry, Cairo University, Giza, Egypt; 2 Oral Radiology, Faculty of Dentistry, Cairo University, Giza, Egypt

**Keywords:** Paediatric dentistry, Dental materials, Restorative dentistry

## Abstract

**Aim:**

Clinical and radiographic evaluation of SDF versus MTA as indirect pulp capping agents in deeply carious first permanent molars.

**Methodology:**

This study was conducted on (30) first permanent molars indicated for indirect pulp capping (IPC) randomly allocated to either SDF or MTA groups (*n* = 15). The molars were finally restored with glass hybrid glass ionomer restoration. Clinical assessment was conducted at 3, 6, 9 and 12 months, while radiographic assessment was performed at 6 and 12 months using predetermined criteria. Data was statistically analyzed.

**Results:**

There was no statistically significant difference between both groups for all assessed clinical and radiographic parameters, including dentin bridge formation, at all follow-up periods. There was no evidence of clinical or radiographic failure in either group.

**Conclusions:**

SDF showed a high success rate compared to MTA and can be considered a promising alternative IPC agent in permanent molars.

## Introduction

The First permanent molar (FPM) plays a critical role in oral function, bearing the highest occlusal load and impacting overall dental health. FPM erupts early in the oral cavity. Therefore, it becomes particularly susceptible to dental decay due to its functional and morphological characteristics [1]. According to existing literature, FPM exhibits the highest incidence of dental caries. Hence, dental practitioners must prioritize prompt treatment of carious lesions in their early stages [2].

If a deep carious lesion is entirely removed from the first permanent molar (FPM), the risk of pulp exposure increases, potentially necessitating root canal therapy [3]. Therefore, Indirect Pulp Capping (IPC) is the preferred treatment for preserving pulp vitality in teeth affected by deep caries without signs of irreversible pulp alterations [3, 4]. The IPC technique has shown favorable clinical and radiographic outcomes [5]. Additionally, IPC is a minimally invasive method for managing deep caries, requiring less time and expense compared to conventional treatments [6]. The American Academy of Pediatric Dentistry (AAPD) recommends the single-step IPC technique over the stepwise approach [5, 7].

Materials used in IPC should ideally have the following properties: adhesion, seal, insolubleness, dimensional stability, non-resorbability, non-toxic, noncarcinogenic, radiopaque, biocompatibility and bioactivity, which no material can fulfill all [8].

For years, Calcium hydroxide (CaOH) has been used for vital pulp therapy, but concerns have arisen due to its solubility, lack of adhesion, and inadequate mechanical properties. This has prompted the search for alternative materials, including mineral trioxide aggregate (MTA). MTA is now considered the gold standard for IPC [9].

Mineral trioxide aggregate has been recommended for IPC by the AAPD [5]. Among the favorable properties of MTA are good biocompatibility, superior sealing ability, low solubility and long-term stability. However, MTA has some drawbacks, including high cost, difficult manipulation, and long setting time [10]. Owing to these drawbacks, the search for ideal IPC material continues.

Among the suggested materials in the literature was Silver diamine fluoride (SDF). SDF is currently used to arrest carious lesions in primary teeth; nevertheless, according to the AAPD, 2023, more research is required to arrest caries lesions in permanent teeth [11]. The Food and Drug Administration currently approves using SDF to arrest carious lesions in permanent teeth [12].

The mechanism of action of SDF in caries arrest is still debatable and is only partially explained. SDF combines silver’s antibacterial properties with fluoride’s remineralizing potential. Also, the SDF products inhibit the proteolytic enzymes responsible for protein degradation and block dentinal tubules. Thus, SDF acts as a desensitizing agent [13].

Few studies regarding SDF use as IPC in permanent teeth were available in the literature. However, SDF use yielded efficient and cost-effective results and was often well-tolerated by dental pulp tissue [14]. SDF also promoted enhanced odontoblastic activity and the production of tertiary dentin [15].

A previous study assessed the remineralizing effectiveness of silver diamine fluoride compared to glass ionomer Type VII and CaOH in mature permanent teeth. After three months, all groups exhibited a nearly identical increase in the percentage of calcium levels [16]. Another research compared the effectiveness of 38% SDF with and without potassium iodide to RMGI in treating deep carious lesions in young permanent molars. There were no statistically significant differences among the groups for secondary caries, postoperative pain, tooth vitality, clinical abscess, radiographic evidence of pulpal pathology, restorations’ marginal adaptability, anatomic form, and surface roughness at all stages for 12 months follow-up [17].

At the time of the study, the available research on SDF use in IPC in permanent teeth was either in-vitro or in-vivo studies measuring histological sections following tooth extraction or dentin samples after reopening the cavity. The current research differs in conducting a randomized clinical trial, measuring clinical and radiographic success, and having a one-year follow-up period. Additionally, this study was intended to benefit the patients by saving first permanent molars with deep carious lesions and the clinicians by comparing the clinical and radiographic outcomes of SDF and MTA when used as materials for IPC in permanent molars. Thus, more convenient options may be recommended for future use. A null hypothesis was adopted, stating that no statistically significant difference exists between the tested materials as indirect pulp capping agents for deeply carious first permanent molars, based on the clinical and radiographic outcomes evaluated.

## Materials and methods

### Study design

The present study is a randomized clinical trial conducted to evaluate the clinical and radiographic outcomes of using SDF versus MTA in IPC in permanent molars. The reporting of this clinical investigation was ensured by adhering to the Consolidated Standards of Reporting Trials (CONSORT) guidelines [18].

### Sample size

In a previous study by Leye Benoist et al. [19], the difference in dentin bridge thickness within the MTA group after 6 months was normally distributed with a standard deviation of 0.11. Suppose the estimated true difference between SDF and MTA means is 0.13. in that case, we will need to study (12) teeth per group to be able to reject the null hypothesis that the population means of the experimental and control groups are equal with probability (power) 0.8. The Type I error probability associated with this test of this null hypothesis is 0.05. The sample size was increased by 25% to compensate for possible dropouts to reach (15) teeth per group. The sample size was calculated using PS Power and Sample for Windows version 3.1.6 using an independent t-test [20].

### Ethical aspects

The Ethics Committee of Scientific Research, Faculty of Dentistry, Cairo University, approved the current research in accordance with the Helsinki Declaration with approval number 14722 [21, 22].

The child’s parent or legal guardian signed a written informed consent.

The study was registered on clinicaltrials.gov with ID number NCT05425368.

### Study setting

The study was conducted in the Pediatric Dentistry and Dental Public Health department, Faculty of Dentistry, Cairo University.

### Eligibility criteria

Vital deeply carious first permanent mandibular molar(s).

### For the children

#### Inclusion criteria

- Showing cooperation and compliance.
- 9–14 years old.

#### Exclusion criteria

- Immunocompromised status or silver allergy.
- Severe periodontal disease or rampant caries.
- Molar incisor hypomineralization.

### For the molars

#### Inclusion criteria

- First permanent mandibular molar(s) with complete root formation and deep occlusal caries extending into dentin.
- Asymptomatic teeth.

#### Exclusion criteria

- Cervical and proximal lesions.
- Any clinical or radiographic signs of irreversible pulp pathologies or pulp necrosis.
- Remaining dentin thickness less than 0.5 mm or more than 2 mm.

#### Randomization and allocation concealment

The eligible teeth were randomly allocated into two groups: Group I, treated with SDF (the intervention), and Group II, treated with MTA (the control). The sequence was generated on a Microsoft Excel sheet (performed by a colleague not involved in the research) and randomized with a 1:1 ratio using (www.random.org). The allocation was concealed in 30 opaque sealed envelopes, each folded eight times and assigned to a group to be followed. Once the cavity preparation was completed, the participant child opened the envelope and handed it to the operator.

### Blinding

The participating children, their legal guardians, one of the radiographic assessors and the statistician were blinded.

#### Baseline clinical examination

**Fig. 1:**
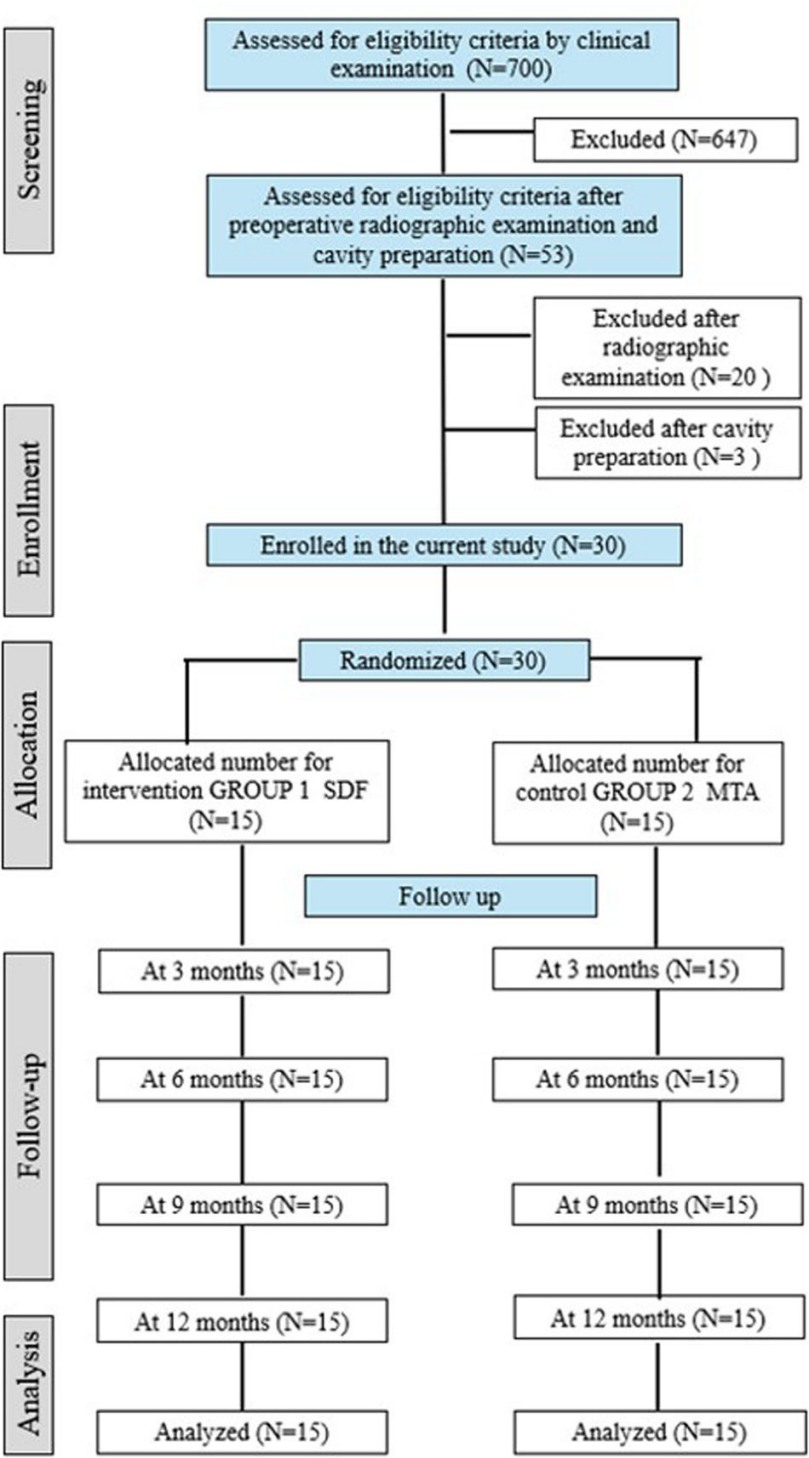
Flowchart of the patients in the current study.

The flowchart in Fig. (1) shows the recruitment of patients in the current study.

- A custom-made diagnostic chart was used, including history taking from the child and the guardian (personal, medical, dental, chief complaint and precise history of pain).
- Clinical examination was divided into two parts.

- Comprehensive soft tissue examination of the chief complaint area to detect the presence of swelling, draining sinus or fistula.
- Hard tissue examination where the suspected tooth was dried and examined to evaluate the degree of carious involvement, the remaining tooth structure, tenderness to percussion (vertical and horizontal) and mobility. The affected tooth was compared with adjacent or contralateral teeth.
- Preoperative photographs were taken as a baseline and in every follow-up visit.

#### Baseline radiographic examination

- A preoperative conventional periapical radiograph was made using a size two digital radiographic sensor (70 kVp, 7 mA, 0.125 s) with the Digora Software to assess carious lesion size and any abnormal radiographic findings.

All children were instructed to maintain good oral hygiene. When indicated, pre-operative dental prophylaxis was recommended.

#### Operative procedure

- Cold pulp testing using ENDO ICE (−50 °C, Tetra fluoro ethane) on a small cotton pellet placed on the buccal surface of the tooth [4].
- Assessment of centric occlusal stops using articulating paper before cavity preparation [23].
- Topical 20% benzocaine gel was applied initially for 2–3 min. This was followed by a subsequent injection of 4% articaine hydrochloride (40.00 mg with 0.01 mg epinephrine) for inferior alveolar nerve block anesthesia.
- Vaseline gel was applied to protect the gingiva, oral mucosa, and circumoral skin from accidental staining by SDF [24].
- Rubber dam isolation followed by cavity preparation using a sterile high-speed carbide bur No. 245 and a high-speed handpiece with water spray to remove enamel caries and gain access to dentin caries [25].
- The operator used 3.5X magnification loupes with an attached extra light source for improved visibility [25].
- Removal of all caries located at the dentin enamel junction or within the outer 2 mm of dentin was done [26].
- The soft infected dentin layer was meticulously removed using a suitably sized sharp spoon excavator. This process relied on assessing the hardness, tactile sensation, and visual appearance of the carious dentin to guide precise removal [27].
- Teeth with unintentional pulp exposure due to gross soft decay were excluded from the study and received root canal treatment followed by stainless steel crown restoration.
- After excavation, the cavity was carefully dried, and then the residual layer was covered with the randomly chosen liner material and manipulated according to the manufacturer’s instructions.

#### GROUP 1 (silver diamine fluoride)

- A drop of SDF (25% Silver, 5.5% Fluoride, 8% Ammonia, Advantage Arrest®, ELEVATE ORAL CARE, LLC, USA) was added to a plastic dappen dish and then applied to the cavity with a micro brush. The SDF was then gently rubbed for 1 min [24, 28].

#### GROUP 2 (mineral trioxide aggregate)

- A spatula was used to dispense an increment of the putty consistency MTA (NeoPUTTY®, Avalon Biomed, Houston, TX USA) on a clean glass slab and then added to the cavity using an MTA applicator.

#### For both groups

- The final restoration utilized glass hybrid glass ionomer filling (EQUIA Forte® HT, © GC CORPORATION, TOKYO, JAPAN). Dentin conditioner was applied to the cavity for 20 s using a micro brush. This was followed by rinsing and gentle drying to maintain a moist surface without desiccation.
- The glass ionomer was applied and contoured using a ball burnisher.
- After allowing 2 min and 30 s for the initial setting, the rubber dam was removed, occlusal interferences were checked with articulating paper, adjusted and confirmed for patient comfort.
- A coat of glass ionomer varnish was applied with a micro brush and light cured [23].
- A customized radiographic stent (with an embedded 5 mm stainless steel wire) was created for each child and utilized with an Extension Cone Paralleling (XCP) dental X-ray film positioning device. This step was done to ensure consistent and accurate standardization of the digital radiographs [29]. The previously mentioned tools were used with a size two digital radiographic sensor (70 kVp, 7 mA, 0.250 seconds) to make the post-operative and follow-up radiographs.
- A follow-up period of 3, 6, 9 and 12 months was planned for the subjects.

### Outcomes

The outcomes measured are illustrated in Table (1).

**Fig. 2:**
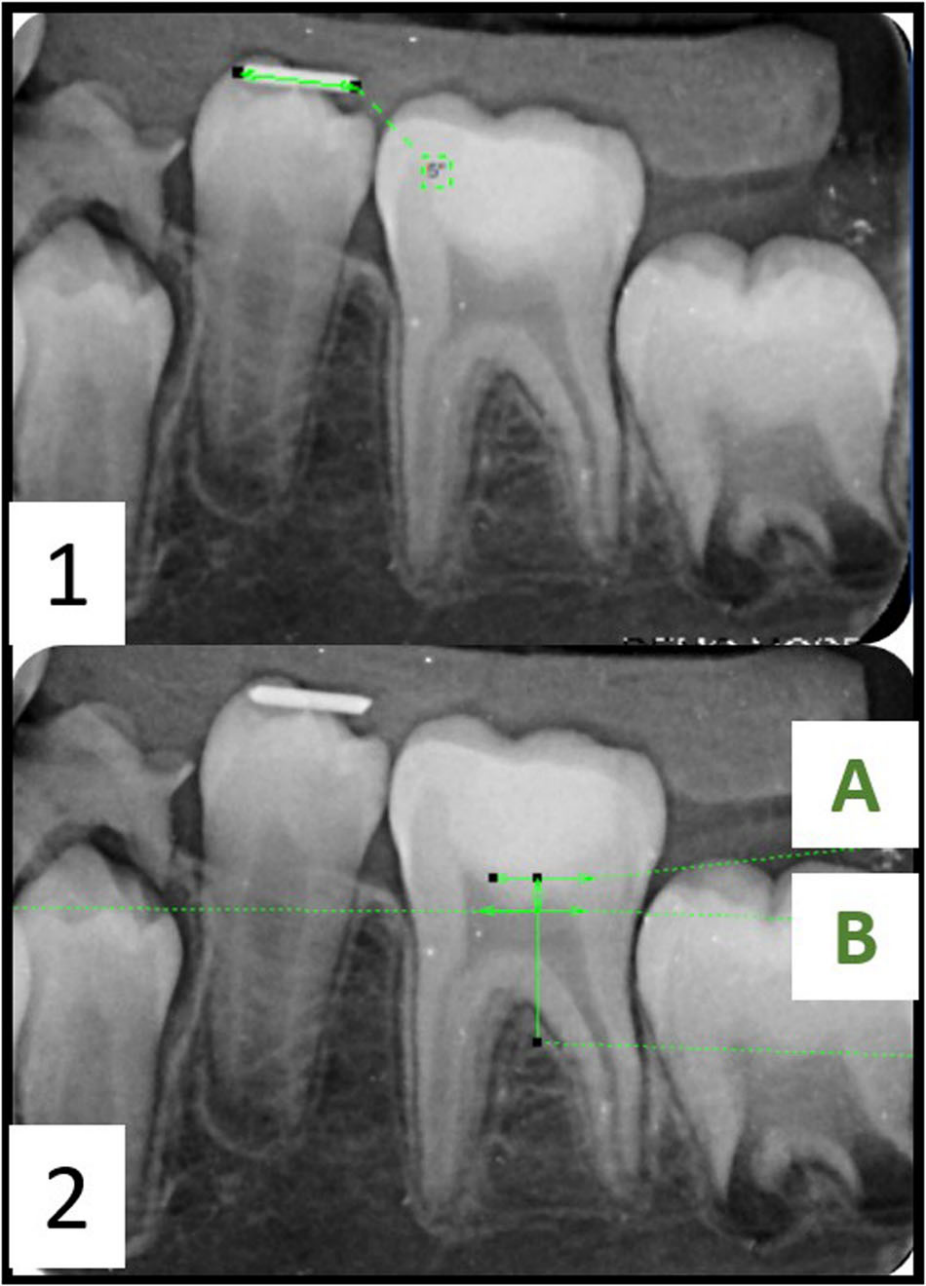
Dentin bridge measurement; (1) Postoperative radiograph showing the wire used in the calibration step, (2) reference lines used in measurement; (A) Reference point representing the most apical point of the restoration, (B) Reference point representing the most apical point in the dentin beneath the restoration corresponding to point A, (A, B) The distance between point A and point B is the line representing the remaining dentin thickness.

- The clinical outcomes were measured in the baseline, 3, 6, 9 and 12 months. The post-operative pain was recorded only once.
- The radiographic outcomes were measured in the baseline, 6 and 12 months.
- Findings were recorded in the custom-made chart.
- All radiographs were evaluated by the same radiographic assessor (blinded) and the principal investigator.
- **Dentin bridge thickness:**
  - The dentin bridge formation was assessed by digital radiographic examination, as shown in Fig. (2) [29]. This was measured in mm using DIGORA software for Windows (2.7 R2, Digora, Orion Corporation, SOREDEX, Medical system, Tuusula, Finland).
  - The radiograph was first calibrated to ensure accurate measurement.
  - Two distinct tangential lines were drawn, passing at two predefined and replicable points (A and B), as shown in Fig. (2).
  - Reference point (A) represented the most apical point of the restoration, while reference point (B) represented the most apical point in the dentin beneath the restoration corresponding to point A.
  - To guarantee accurate measurement of the distance between the two lines, a 90° angle was first constructed between the two lines.
  - A third line was drawn connecting the two previously mentioned lines and coinciding with the 90° angle. This third line from point (A) to point (B) denotes the thickness of the remaining dentin.

**Table 1:** Clinical and radiographic outcomes measured in the current study.

	Name of outcome		Outcome measuring device	Outcome measuring unit
Primary outcome:	Clinical success	Absence of post-operative pain	Asking the patient and the guardian	Binary (present/absent)
		Absence of spontaneous pain	Asking the patient and the guardian	Binary (present/absent)
		Absence of pain on percussion	Percussion test using the back of a dental mirror	Binary (present/absent)
		Absence of Swelling	Visual examination	Binary (present/absent)
		Absence of Sinus or fistula	Visual examination	Binary (present/absent)
		Pulp sensibility test	Thermal (cold Ice) test	Binary (present/absent)
Secondary outcomes:	Radiographic Success	Absence of any adverse radiographic finding (Periodontal membrane space widening, bone resorption, pulp stones	Digital Radiographic examination	Binary (present/absent)
	Dentin bridge thickness		Digital Radiographic examination	mm

**Fig. 3:**
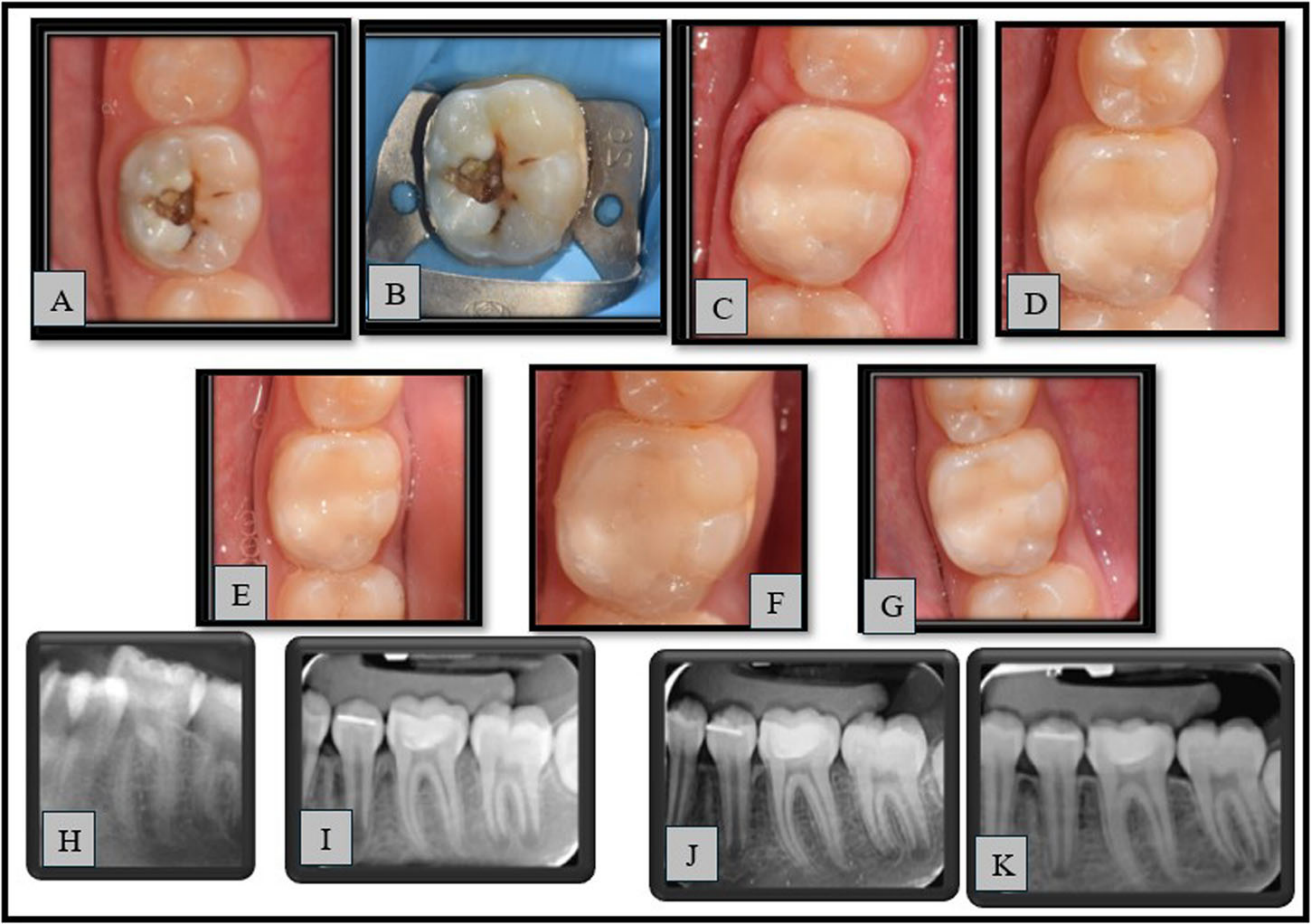
Representative case of GROUP 1 (SDF) showing clinical photographs and radiographs. **A**–**G**: Clinical photographs (**A**—pre-operative, **B**—Preoperative with rubber dam, **C**—Immediate post-operative, **D**—3 months, **E**—6 months, **F**—9 months, **G**—12 months), **H**–**K**: Radiographs (**H**—pre-operative, **I**—Immediate post-operative, **J**—6 months, **K**—12 months).

**Fig. 4:**
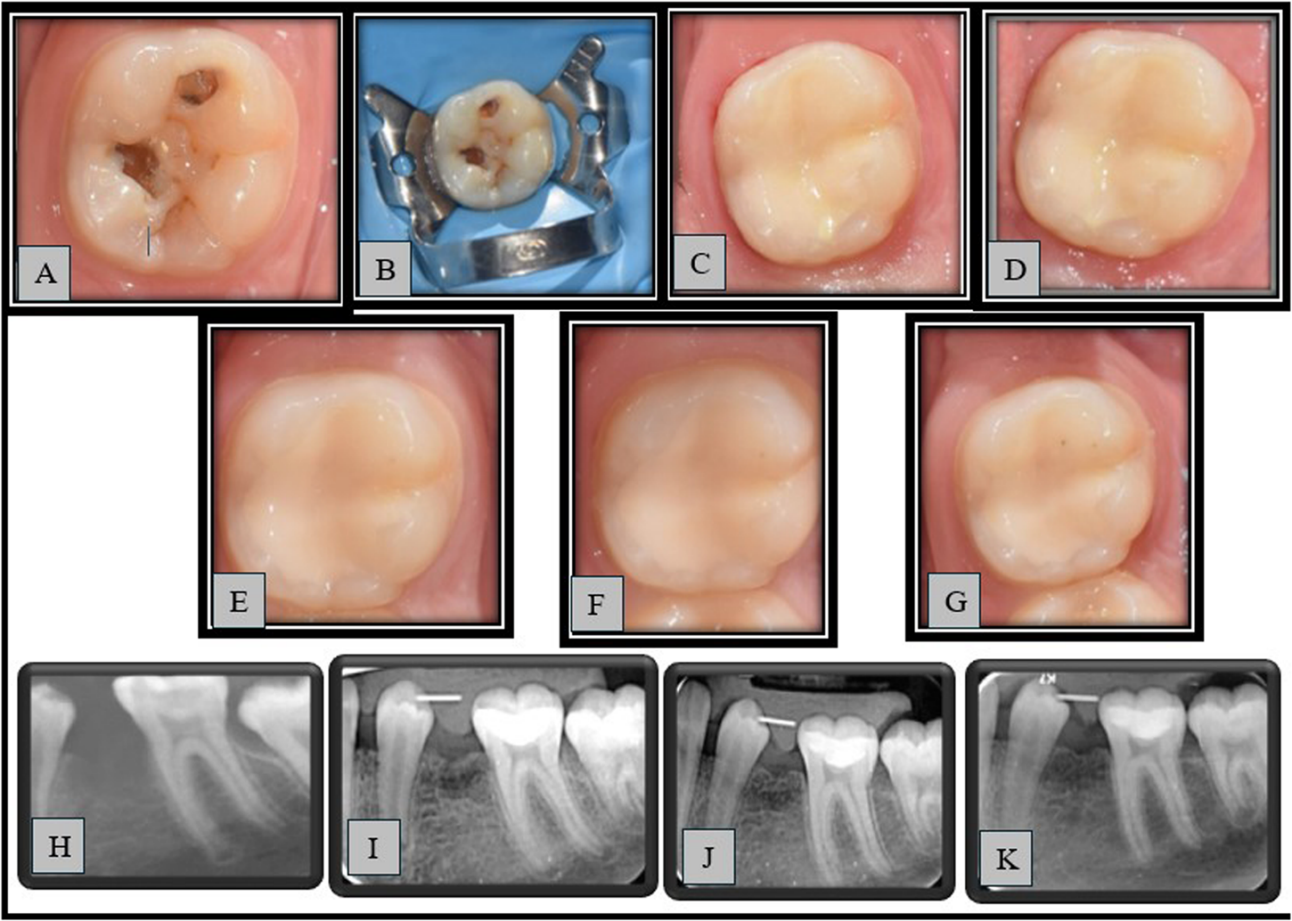
Representative case of GROUP 2 (MTA) showing clinical photographs and radiographs. **A**–**G**: Clinical photographs (**A**—pre-operative, **B**—Preoperative with rubber dam, **C**—Immediate post-operative, **D**—3 months, **E**—6 months, **F**—9 months, **G**—12 months), **H**–**K**: Radiographs (**H**—pre-operative, **I**—Immediate post-operative, **J**—6 months, **K**—12 months).

Representative cases from group (1) and group (2) are shown in Figs. (3) and (4) respectively.

### Statistical analysis

MedCalc software, version 22 (windows), was used to analyze the data. Frequency and percentage were used to describe categorical data. A chi-square test with a statistical significance level set at (*P* ≤ 0.05) was used for intergroup comparisons between interventions. Cochran’s Q test, followed by multiple comparisons, was used for intragroup comparison within each intervention. The clinical significance was assessed by Relative risk. Kolmogorov Smirnov and Shapiro-Wilk tests were used to assess continuous data for normality. Continuous data showed normal distribution and were described using mean and standard deviation. An independent t-test with a statistical significance level set at (*P* ≤ 0.05) was used for intergroup comparison of continuous data. Repeated measures ANOVA was used for intragroup comparison.

Cohen’s kappa coefficient was used to calculate inter-observer agreement. The confidence level was established at 95% with a statistical power of 80%, and all tests were conducted using a two-tailed approach.

### Reference software

Endnote desktop software (EndNote 21.3 bld (17918), Apryse SDK©) was used for managing references.

## Results

This study was conducted on (30) first permanent molars randomly allocated to the intervention and the comparator arms (*n* = 15). After 12 months, all participants completed the follow-up with a 100% retention rate. The mean age of the participants in the current trial was 10.75 ± 1.05 years. The mean age within the intervention group was 10.73 ± 1.26 years, while within the comparator group, the mean age was 10.76 ± 0.83 years. There was no statistically significant difference between both groups regarding age (*P* = 0.946). Regarding gender, 13 boys and 17 girls were in the current study, and there was no statistically significant difference between both groups (*P* = 0.7172). Regarding the side of the mandible, there were 12 molars on the right side and 18 molars on the left side. There was no statistically significant difference between both groups regarding the side of the mandible (*P* = 1.0000).

After 12 months, both groups have shown a 100% clinical success rate. The clinical outcomes are illustrated in Table (2). There was no risk for swelling or sinus and fistula in GROUP 1 compared to GROUP 2 at all follow-up periods (RR = 1.0000 (95% 0.0211 to 47.383; *P* = 1.0000)). There was no evidence of swelling or sinus and fistula in both groups (with no statistically significant difference between both groups) at baseline 3, 6, 9 and 12 months.

**Table 2:** Clinical outcomes within each group.

Clinical Outcomes	Follow-up	GROUP 1		GROUP 2		*P*-value
		No	Yes	No	Yes	
Post operative pain	Baseline	14 (93.3%)	1 (6.7%)	14(93.3%)	1 (6.7%)	*P* = 1.0000
Spontaneous pain	Baseline	15 (100%)	0 (0%)	15 (100%)	0 (0%)	*P* = 1.0000
	3 months	15 (100%)	0 (0%)	15 (100%)	0 (0%)	*P* = 1.0000
	6 months	15 (100%)	0 (0%)	15 (100%)	0 (0%)	*P* = 1.0000
	9 months	15 (100%)	0 (0%)	15 (100%)	0 (0%)	*P* = 1.0000
	12 months	15 (100%)	0 (0%)	15 (100%)	0 (0%)	*P* = 1.0000
	*P*-value	*P* = 1.0000		*P* = 1.0000		RR = 1.0000
Pain on percussion	Baseline	15 (100%)	0 (0%)	15 (100%)	0 (0%)	*P* = 1.0000
	3 months	15 (100%)	0 (0%)	15 (100%)	0 (0%)	*P* = 1.0000
	6 months	15 (100%)	0 (0%)	15 (100%)	0 (0%)	*P* = 1.0000
	9 months	15 (100%)	0 (0%)	15 (100%)	0 (0%)	*P* = 1.0000
	12 months	15 (100%)	0 (0%)	15 (100%)	0 (0%)	*P* = 1.0000
	*P*-value	*P* = 1.0000		*P* = 1.0000		RR = 1.0000
Pulp sensibility test	Baseline	14 (93.3%)	1 (6.7%)	15 (100%)	0 (0%)	*P* = 0.3173
	3 months	14 (93.3%)	1 (6.7%)	15 (100%)	0 (0%)	P = 0.3173
	6 months	14 (93.3%)	1 (6.7%)	15 (100%)	0 (0%)	*P* = 0.3173
	9 months	14 (93.3%)	1 (6.7%)	15 (100%)	0 (0%)	*P* = 0.3173
	12 months	14 (93.3%)	1 (6.7%)	15 (100%)	0 (0%)	*P* = 0.3173
	*P*-value	*P* = 1.0000		*P* = 1.0000		RR = 3.0000

After 12 months, both groups showed a 100% radiographic success rate. The radiographic outcomes are illustrated in Table (3). The calculated Cohen’s Kappa coefficient for both assessors was found to be 1.

**Table 3:** Radiographic outcomes within each group.

Clinical Outcomes	Follow-up	GROUP 1		GROUP 2		*P*-value
		No	Yes	No	Yes	
Periodontal membrane space widening	Baseline	15 (100%)	0 (0%)	15 (100%)	0 (0%)	*P* = 1.0000
	3 months	15 (100%)	0 (0%)	15 (100%)	0 (0%)	*P* = 1.0000
	6 months	15 (100%)	0 (0%)	15 (100%)	0 (0%)	*P* = 1.0000
	9 months	15 (100%)	0 (0%)	15 (100%)	0 (0%)	*P* = 1.0000
	12 months	15 (100%)	0 (0%)	15 (100%)	0 (0%)	*P* = 1.0000
	*P*-value	*P* = 1.0000		P = 1.0000		RR = 1.0000
Bone resorption	Baseline	15 (100%)	0 (0%)	15 (100%)	0 (0%)	*P* = 1.0000
	3 months	15 (100%)	0 (0%)	15 (100%)	0 (0%)	*P* = 1.0000
	6 months	15 (100%)	0 (0%)	15 (100%)	0 (0%)	*P* = 1.0000
	9 months	15 (100%)	0 (0%)	15 (100%)	0 (0%)	*P* = 1.0000
	12 months	15 (100%)	0 (0%)	15 (100%)	0 (0%)	*P* = 1.0000
	*P*-value	*P* = 1.0000		*P* = 1.0000		RR = 1.0000
Pulp stones	Baseline	15 (100%)	0 (0%)	15 (100%)	0 (0%)	*P* = 1.0000
	3 months	15 (100%)	0 (0%)	15 (100%)	0 (0%)	*P* = 1.0000
	6 months	15 (100%)	0 (0%)	15 (100%)	0 (0%)	*P* = 1.0000
	9 months	15 (100%)	0 (0%)	15 (100%)	0 (0%)	*P* = 1.0000
	12 months	15 (100%)	0 (0%)	15 (100%)	0 (0%)	*P* = 1.0000
	*P*-value	*P* = 1.0000		*P* = 1.0000		RR = 1.0000

Regarding the dentin bridge thickness shown in Table (4), both groups showed a statistically significant increase in dentin bridge thickness after 12 months. There was no statistically significant difference between both groups regarding the mean difference in dentin bridge formation (12 months-baseline), with a mean difference of −0.09 ± 0.14 showing a small effect size (*P* = 0.0908).

**Table 4:** Mean and standard deviation for dentin bridge thickness for the intergroup comparison between groups within each follow-up and intragroup comparison within each group between different follow-up periods.

Follow-up	GROUP 1		GROUP 2		Mean difference		*P*-value
	Mean	SD	Mean	SD	Mean	SD	
Baseline	1.25^c^	0.37	0.91^c^	0.38			
6 months	1.40^b^	0.45	0.99^b^	0.37			
12 months	1.54^a^	0.44	1.10^a^	0.32			
Mean difference at 6 months (6 months-baseline)	0.15	0.58	0.08	0.54	0.07	0.79	*P* = 0.7131
Mean difference at 12 months (12 months-baseline)	0.30	0.13	0.20	0.15	0.09	0.14	*P* = 0.0908
*P*-value	*P* < 0.001^*^		*P* < 0.001^*^				

Superscript letters (a, b, c) indicate statistically significant differences between time points within the same group (Group 1 or Group 2).

Means that do not share a letter vertically are statistically significant.

^*^denotes statistically significant.

## Discussion

Recent approaches to managing deep carious lesions focus on preserving pulp vitality. The present study selected the single-step IPC technique [30]. In this approach, infected dentin was removed, leaving the affected dentin, which can undergo remineralization and allow the odontoblasts to generate reactionary dentin [16].

Mineral trioxide aggregate, the current gold standard for IPC in permanent teeth, was chosen for the control group [10]. However, owing to the previously mentioned drawbacks of MTA, more studies were needed to search for an ideal pulp capping agent. SDF was chosen as the intervention since it is nowadays used to arrest caries lesions in primary teeth. Nevertheless, according to the AAPD, 2023, more research is needed on using SDF to arrest carious lesions in permanent teeth [11]. Also, limited data was available in the literature regarding using SDF in IPC in permanent teeth.

In the current research, complete blinding of the operator was not feasible due to the apparent difference in the capping materials used. This problem was approached by semi-blinding the operator, i.e., not knowing the capping material until finishing the cavity preparation (the most crucial step in IPC). The treatment of both groups was standardized using objective outcomes as applicable and an independent radiographic outcome assessor [31].

The present study included 13 boys and 17 girls, and there was no statistically significant difference between both groups regarding gender. A systematic review stated that female children were 1.14 times more likely to have poor oral health than male children [32].

No single failure report was evident regarding both groups’ overall clinical success rate after 12 months of follow-up. For GROUP 1, this is comparable to the results of a similar study by Baraka et al. [17] who reported a 97.2% success rate for SDF in IPC after 12 months. For GROUP 2, this is similar to previous studies, which reported high success rates for MTA in IPC [33, 34]. The high success rate of SDF can be explained by its antibacterial action, remineralizing potential and desensitizing effect [13]. The high success rate of MTA can be linked to its good sealing ability and bioactivity [10]. Also, the IPC approach for deep caries management has a high success rate and is based on some other factors rather than the type of material used, among which is the good coronal seal and correct case selection [5].

Considering the spontaneous post-operative pain, this was assessed by a telephone call one week after the treatment to avoid repeated school absences*.* Two patients reported post-operative pain, one in each group, in the form of sensitivity. The guardians were then instructed to bring the children to the hospital if the pain recurred. However, the sensitivity subsided during later follow-up visits. This sensitivity may be attributed to the inherent acidity of glass ionomer, particularly the acid conditioning of the cavities, as explained by El-Bialy et al. [23].

Considering the pain on percussion, all cases in both groups showed no pain on percussion, which agrees with the results of previous studies [16, 35]. This was also evident regarding the absence of swelling, sinus or fistula in the current research, which is similar to the findings of Sinha et al. [16].

Regarding the response to the sensibility test, during the 12-month follow-up period, there was no statistically significant difference among each group or between both groups, which is close to the results of previous studies [17, 36]. Meanwhile, one patient in GROUP 1 failed to respond to the sensibility test since the baseline and during the 12-month follow-up. The patient reported clinical and radiographic success in all other examined parameters. This can be explained by the fact that some children could be less reliable in responding to the sensibility test, as mentioned in previous literature [37].

The radiographic success rate in the current study was 100% in both groups, which correlates with previous studies [16, 38]. Also, periodontal membrane space widening, bone resorption, and pulp stones were not evident in the current research findings, which agrees with the results of a previous study [38]. The calculated Cohen’s Kappa coefficient was 1, indicating perfect agreement between the radiographic assessors.

Regarding dentin bridge thickening, there was a statistically significant increase in dentin bridge thickness in both groups after the 12-month follow-up period with 0.30 (±0.13) mm and 0.20 (±0.15) mm increase, respectively. This was unlike the results reported by a previous study [29]. This disagreement may be attributed to the use of different IPC materials. However, the current study’s results of dentin bridge thickening came in accordance with the measurements of previous research [19].

For the intergroup comparison, there was no statistically significant difference between both groups regarding dentin bridge formation after 12 months. This can be attributed to the remineralizing potential of SDF and the bioactive potential of MTA, as explained in previous literature [10, 16]. This finding was compared to a recent study that reported no variations among the groups regarding the quality and quantity of tertiary dentin formation following IPC by SDF compared to glass ionomer cement (using CBCT) [38].

The comparison between the two groups revealed no statistically significant difference in the clinical and radiographic parameters assessed; therefore, the current study failed to reject the null hypothesis.

A limitation faced during the current study was the radiographic stent. Although it is recommended in the literature, the precise reproducibility of intraoral periapical radiographs using the stent represented a challenge due to the ongoing growth and dynamic nature of the teeth during the mixed dentition stage. Also, the sensibility test was less reliable in children, with some probability of having a false negative or false positive response. Additionally, the patients were followed up for one year only.

## Conclusions

Silver diamine fluoride showed a high success rate as MTA when used as a material for IPC in permanent teeth. SDF can be considered a promising alternative to MTA in IPC in permanent molars, with no adverse effects on dental pulp tissue for a one-year follow-up period.

## Data Availability

Data supporting the findings of this research will be made available upon reasonable request.
